# Familial Mediterranean Fever: An Autoinflammatory Genetic Disorder

**DOI:** 10.7759/cureus.69856

**Published:** 2024-09-21

**Authors:** Rafaela Lopes Freitas, Nídia Pereira, Adelina Pereira

**Affiliations:** 1 Internal Medicine Service, Pedro Hispano Hospital, Matosinhos Local Health Unit, Matosinhos, PRT

**Keywords:** auto-imune diseases, autoinflammatory diseases, familial mediterranean fever, monogenic syndromes, rare genetic diseases

## Abstract

A man in his 30s with a past medical history of fever episodes of unknown origin associated with abdominal and chest pain, arthralgias, and two episodes of aseptic meningitis, beginning at teenage, presented at the emergency department with similar symptoms and tinnitus with one week of evolution. A physical examination revealed left peripheral facial paresis and bilateral sensorineural deafness.

From the etiological investigation, numerous tests were conducted to rule out infectious, paraneoplastic, and immune disorders, all of which yielded unremarkable results. He began a high dose of corticosteroids, leading to complete clinical recovery.

Monogenic autoinflammatory syndrome disease was suspected. A genetic test confirmed the diagnosis of familial Mediterranean fever. He began taking colchicine daily without any complications.

To avoid future complications, the authors aim to emphasize the importance of recognizing this rare cause of fever.

## Introduction

The concept of autoinflammatory disease was introduced to differentiate a group of inflammatory autosomal-dominant disorders from autoimmune diseases, which are characterized by self-targeting immune responses. Autoinflammatory diseases, in contrast, primarily involve dysregulation of the innate immune system without the involvement of adaptive immunity or autoantibodies.

Autoinflammatory syndromes are rare and nowadays are divided into monogenic, polygenic, and undifferentiated. Specifically, monogenic autoinflammatory diseases can be classified by their type of inflammatory mechanism, such as changes in the inflammasome (for example, increased production of interleukin 1 (IL-1)); mediation by nuclear factor kappa-light-chain enhancer of activated B cells (NF-KB); modified interferon (IFN); and other pathogenic pathways. The common feature of these illnesses is the presence of self-limited, periodic, or recurrent episodes of inflammation [1].

In fact, familial Mediterranean fever (FMF) is the most common autoinflammatory disorder worldwide [2,3]. It is classically inherited as an autosomal recessive pattern characterized by mutations, mainly missense types, located on chromosome 16 of the MEFV gene [4-6]. This gene encodes a pyrin protein that is expressed in neutrophils, monocytes, dendritic cells, and serosa’s fibroblasts (such as the pleura, peritoneum, synovial, and derma). As a consequence, the defective pyrin induces an exacerbated inflammatory response that results from uncontrolled IL-1 secretion.

FMF is common among people of eastern Mediterranean origin, with an estimated prevalence of 1:500 to 1:1000 in endemic areas [3], yet there is a lack of demographic and epidemiologic data among other populations.

The diagnosis is based on clinical evaluation according to classification criteria, such as Livneh et al. [7]. However, genetic mutation analysis is helpful [8].

Its presentation includes episodes that are characterized by self-limited recurrent fever episodes, serositis, synovitis, and high levels of inflammatory reactants [9,10] with asymptomatic intervals in between. Neurologic manifestations such as aseptic meningitis [11-13] and cochlear involvement [14] were described in FMF patients.

An accurate differential diagnosis is mandatory to exclude infective agents and neoplastic, autoimmune, and metabolic diseases. As a consequence, frequently there is a delay between the first signs/symptoms and the eventual diagnosis.

Regarding treatment, the mainstay therapy is daily colchicine [1], although, in non-responders or intolerants, it is necessary to use biological drugs such as anti-IL1, anti-IL6, or anti-tumor necrosis factor agents. The goals of the treatment are remission of symptoms, prevention of recurrence, normalization of inflammatory markers, and prevention of complications, of which secondary amyloidosis is the most devastating. 

In conclusion, this case report underscores the importance of a comprehensive and multidisciplinary approach in diagnosing complex medical conditions. Despite extensive evaluations and multiple misdiagnoses over several years, the integration of a holistic view of the patient's symptoms ultimately led to the identification of a rare, multi-systemic cause of fever in adults. This highlights the need for continued vigilance and thorough assessment in the diagnosis of atypical presentations of fever, emphasizing that an exhaustive evaluation may be crucial for accurate diagnosis and effective management.

## Case presentation

A man in his 30s presented at the emergency department with recurrent episodic fever mostly associated with abdominal, chest, and/or joint pain.

As a matter of fact, since he was 13 years old, he started having recurrent fever attacks of unknown origin, lasting one to five days, at least one episode per year, with no identified trigger. These episodes were associated with sterile serositis (affecting pericardium once, pleura twice, and peritoneum at least two times), arthralgias occasionally with arthritis affecting large joints, and in two episodes’ aseptic meningitis was documented. Concurrently, leukocytosis and increased acute phase reactants were documented. The common feature among these episodes was its self-limited course, the favorable response to corticosteroids, and the normalization of analytic study in between.

This complex presentation led to several admissions at the hospital and consequently, extensive studies to rule out other differential diagnoses. Diagnosis was never reached.

**Figure 1:**
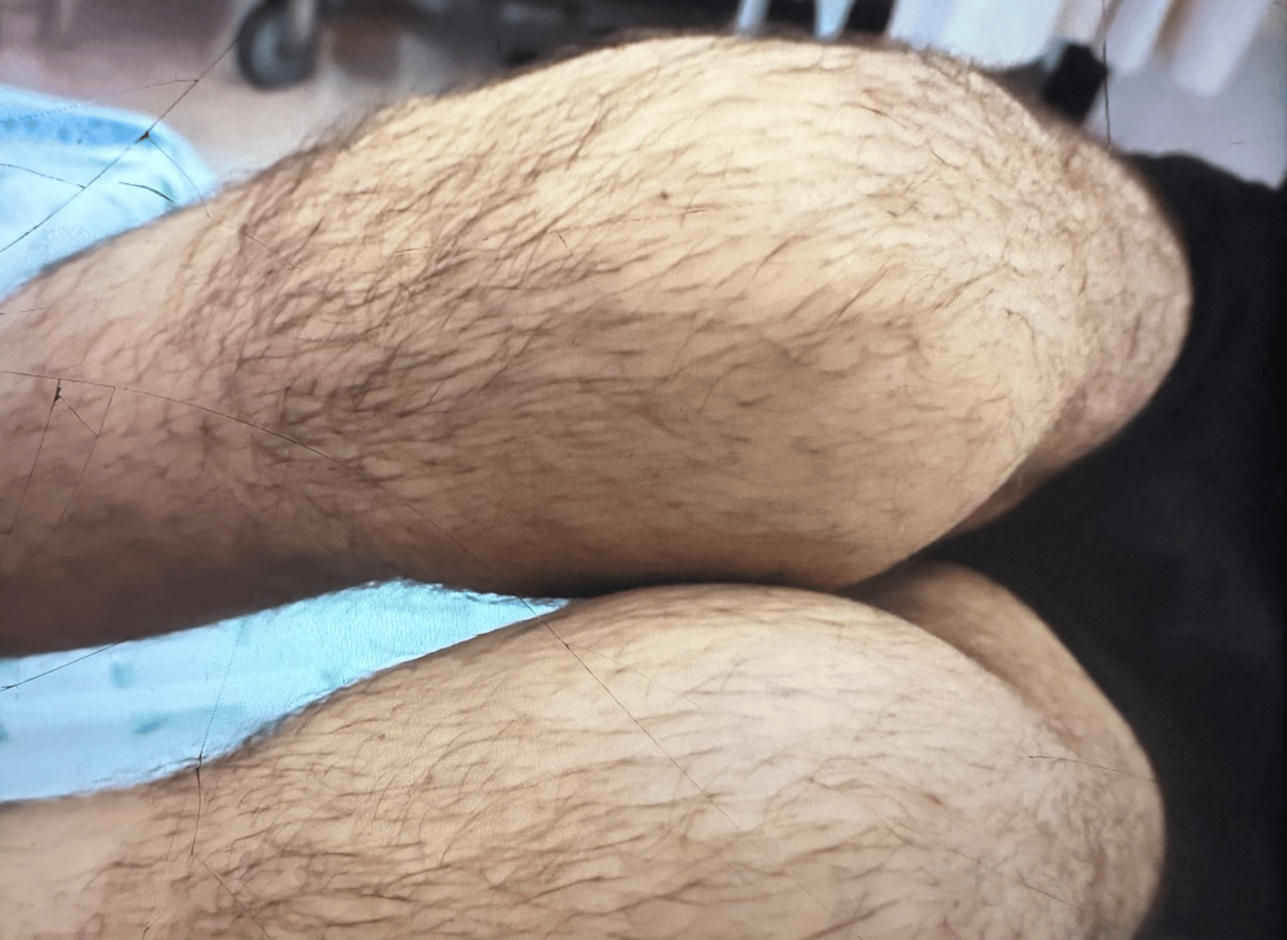
Bilateral knee synovitis

This time the patient presented with fever, asthenia, epigastric pain, knee joint swelling (Figure 1), ankle and hip arthralgias, and bilateral tinnitus with one week of evolution.

**Figure 2:**
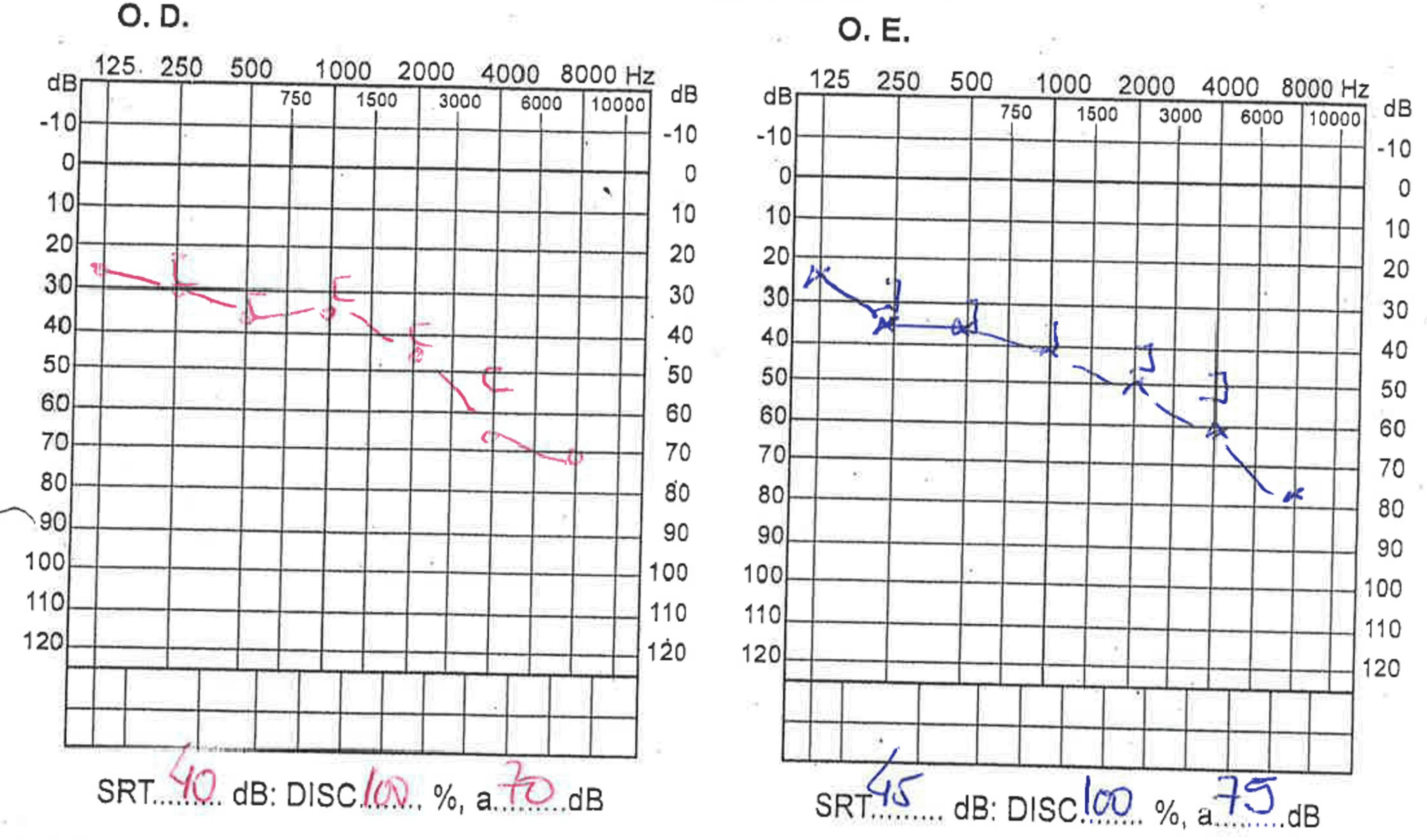
Audiogram showing bilateral sensorineural deafness

Physical examination showed fever (39 ºC), left peripheral facial paresis (House-Brackman stage III), and bilateral sensorineural deafness confirmed by audiogram (Figure 2). There were no other notable findings.

He started a high dose of corticosteroids (1 mg/kg/day of prednisolone) mostly to treat facial paresis with complete clinical resolution in six days.

As far as possible, infectious, paraneoplastic, and immune/inflammatory diseases were excluded.

Tests performed to determine the etiology included complete blood count; routine biochemistry; thyroid function tests; urinalysis; viral serology (human immunodeficiency virus 1 and 2, hepatitis B virus, hepatitis C virus, herpes simplex virus 1 and 2, varicella zoster virus, cytomegalovirus, Epstein-Barr virus) and other serologies (such as Rickettsia, Borrelia, Brucella, Salmonella, Coxiella burnetti); Quantiferon; blood, urine, and spine fluid cultures; peripheral blood and urine immunophenotyping; immunology (antinuclear antibody, anti-double stranded DNA (with Farr radioimmunoassay and Crithidia luciliae indirect immunofluorescence test), extractable nuclear antigen test, antineutrophil cytoplasmic antibodies, rheumatoid factor, anti-cyclic citrullinated peptide, immunoglobulins, cryoglobulins, HLA-B51), all unremarkable.

The echocardiogram was unremarkable. The cerebral-cervico-toraco-abdomino-pelvic computed tomography with angiography only showed mild hepatosplenomegaly.

The only positive findings were the elevated white blood cell count of 13.000/mm3 (normal range 4.000-10.000), erythrocyte sedimentation rate of 56 mm/h (normal range 0-20), C-reactive protein level of 241 mg/L (normal range < 0.5), ferritin 350ng/mL (normal range 12-300), and haptoglobin of 2.5 g/L (normal range of 0.5-2.2) (Table 1). 

**Table 1:** Laboratory test results

Laboratory data	Test value	Normal range
Hemoglobin	12.6 g/dL	13-18 g/Dl
White blood cell count	13.100/uL	4.000-10.000/uL
Platelet count	218/uL	150-400/uL
C-reactive protein	241 mg/L	<0.5 mg/L
Erythrocyte sedimentation rate	81 mm/1h	0-20 mm/1h
Ferritin	350 ng/mL	12-300 ng/mL
Haptoglobin	2.5 g/L	0.5-2.2 g/L
Creatine	0.9 mg/dL	0.7-1.3 mg/dL
Urea nitrogen	22 mg/dL	19-44 mg/dL
Sodium	137 mEq/L	135-135 mEq/L
Potassium	4.1 mEq/L	3.4-5.1 mEq/L
Aspartate transaminase	55 U/L	5-34 U/L
Alanine transaminase	62 U/L	<55 U/L
Alkaline phosphatase	122 U/L	12-64 U/L
Gamma-glutamyl transferase	147 U/L	40-150 U/L
Total bilirubin	0.6 mg/dL	0.2-1.2 mg/dL
Lactate dehydrogenase	168 U/L	125-220 U/L
Creatinine kinase	9 U/L	30-200 U/L
Aldolase	3.7 U/L	0-7.6 U/L

A genetic test was carried out to search for monogenic autoinflammatory diseases with a panel of 95 genes. A heterozygotic MEFV mutation in exon 10 was detected - NM_000243.3(MEFV): c.2084A>G (p.Lys695Arg).

The patient was started on daily colchicine (0.03 mg/kg/day) with good tolerance and, at the same time, a gradual reduction of corticosteroids until suspension in the following month.

**Figure 3:**
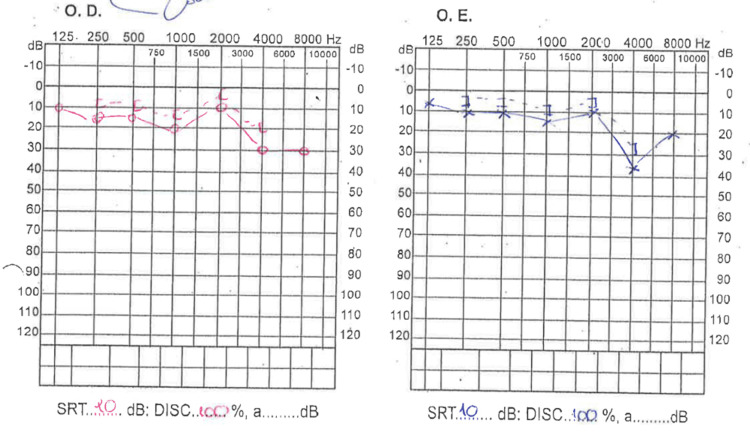
An audiogram showing improvement after the beginning of treatment

After two years of follow-up, there were no other similar episodes or hospital admissions; bilateral neurosensory deafness mildly improved (Figure 3); and there were no other known complications.

## Discussion

The authors present a case of a man with an autoinflammatory disease, most likely to be FMF with some atypia.

Actually, FMF diagnosis might be difficult not only because it exhibits sundry clinical manifestations mimicking other conditions leading to misdiagnosis but also because the majority of MEFV variants are classified as unknown significance [15].

Since 1967, when Sohar et al. [16] proposed the first diagnostic criteria, several classifications have appeared over time. Presently the most used and accepted classification is Livneh from 1997.

Concerning clinical presentation, in this particular case, the patient has more than one major criteria and more than two minor criteria, namely, the age of presentation (< 20 years old), the typical clinical signs and symptoms (such as the self-limited periodic fever > 38ºC, serositis, and arthritis), the spontaneous remission, and the asymptomatic intervals between crises. Even though they are not part of the criteria, the favorable response to colchicine and the presence of hepatosplenomegaly, which is reported in approximately 25% of the patients generally due to inflammation [17], are also some findings that support the diagnosis.

Nevertheless, it might be argued that the episodes of meningitis in this case are unrelated to FMF. As a matter of fact, neurologic manifestations in patients with FMF are rarely reported and not widely recognized as a manifestation. In this case, our patient didn’t have other neurologic abnormalities other than those related to meningeal irritation, and there were no abnormal cells in the spinal fluid or history of trauma. Thus, the most likely explanation is that these episodes of aseptic meningitis were secondary to his condition, given the fact that they appeared at the same time as the other symptoms and had a benign course.

Moreover, the authors questioned whether hearing loss through bilateral neurosensory deafness and facial palsy could be related to FMF. Reassessing current literature, few studies have been performed to investigate hearing loss in FMF patients. The most recent one, from Seza Ozen [18], supported the cochlear involvement in FMF mostly during attacks, probably due to the inflammatory tissue injury process, in which the pathophysiologic mechanisms are not well known yet. With respect to peripheral facial palsy, to the best of our knowledge, there is only one case report [19] that associates this disorder with FMF; hence, the authors believe, based on these observations and the lack of data, that the coexistence of FMF and peripheral facial palsy is not specific.

Regarding genetics, our patient has a heterozygous mutation. It is now known that in 20-40% of FMF cases, there is only one allele mutated [20] and the individual presents classical symptoms, which means that it behaves like an autosomal dominant pattern. In fact, a recent study shows that the genotype-phenotype correlation is incomplete and therefore still not yet well understood [15].

Despite the fact that this was a complex case, the authors alert that there was an alarming gap of more than twenty years between the beginning of symptoms and the formal diagnosis, which according to the reported literature is twice the average time. Probably the clinicians may not consider this disease due to the lack of specific symptoms, the relapsing-remitting pattern, and its rarity, especially in non-ethnic populations.

Certainly, the most disruptive consequence is the development of amyloidosis, which was not confirmed in this case. The main FMF treatment is colchicine, not only because it decreases the number and intensity of acute painful attacks, but it also prevents amyloidosis by reducing subclinical inflammation. 

To conclude, despite the improvement in the disease knowledge, there are still many unsolved questions, mainly in the atypical presentations.

## Conclusions

Familial Mediterranean fever is characterized by self-limiting episodes of fever, polyserositis, and elevated inflammatory markers. FMF should be suspected in all types of patients with such relapsing-remitting, nonspecific symptoms. Genetic testing is a crucial tool to identify these patients. Clinicians should be aware of autoinflammatory syndromes in order to avoid delayed diagnosis, delayed treatment, and serious morbidity due to their complications.
